# Insulin receptor substrate-1 (IRS-1) mediates progesterone receptor-driven stemness and endocrine resistance in oestrogen receptor+ breast cancer

**DOI:** 10.1038/s41416-020-01094-y

**Published:** 2020-11-04

**Authors:** Amy R. Dwyer, Thu H. Truong, Carlos Perez Kerkvliet, Kiran V. Paul, Peter Kabos, Carol A. Sartorius, Carol A. Lange

**Affiliations:** 1 Masonic Cancer Center, University of Minnesota, Minneapolis, MN 55455 USA; 2 Division of Medical Oncology, Department of Medicine, University of Colorado Anschutz Medical Campus, Aurora, CO 80045 USA; 3 Department of Pathology, University of Colorado Anschutz Medical Campus, Aurora, CO 80045 USA; 4 Departments of Medicine (Division of Hematology, Oncology, and Transplantation) and Pharmacology, University of Minnesota, Minneapolis, MN 55455 USA

**Keywords:** Cell biology, Breast cancer

## Abstract

**Background:**

Progesterone receptors (PR) are potent modifiers of endocrine responses. In aberrant signalling cancer contexts, phosphorylation events dramatically alter steroid hormone receptor action.

**Methods:**

The transcriptomes of primary tumours and metastases in mice harbouring ER+ breast cancer patient-derived xenografts (PDXs) were analysed following single-cell RNAseq. In vitro assays were employed to delineate mechanisms of endocrine resistance and stemness.

**Results:**

A 16-gene phospho-Ser294 PR (p-PR) signature predicted poor outcome in ER+ breast cancer. Relative to primary PDX tumours, metastatic lesions expressed abundant p-PR and exhibited an activated PR gene programme with elevated expression of *PGR* and *IRS-1*. Breast cancer models of activated PR lost the expression of IGF1R and acquired insulin hypersensitivity with tamoxifen insensitivity. Activated p-PR+ breast cancer cells formed increased tumourspheres with enlarged ALDH+ and CD24−/CD44 populations. E2 induced PR/IRS-1 interaction and exchange of IGF1Rβ for IRS-1 in p-PR-containing transcriptional complexes. Inhibition of IRS-1 or IR and inducible IRS-1 knockdown reduced tumourspheres. Endocrine-resistant models of luminal B breast cancer induced p-PR in 3D cultures and required PR and IRS-1 for tumoursphere formation.

**Conclusions:**

Phospho-PR-B cooperates with IRS-1 to promote outgrowth of endocrine-resistant and stem-like breast cancer cells. Targeting phospho-PR/IRS-1 crosstalk may block the emergence of endocrine resistance.

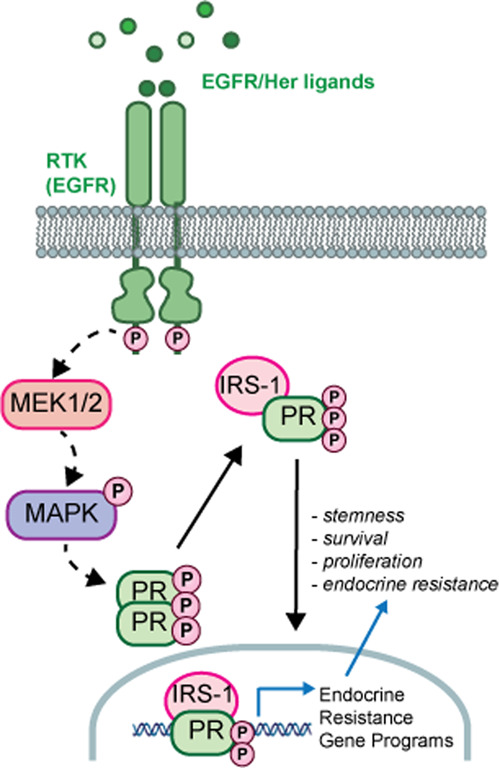

## Background

Breast cancer is the most common malignancy among women and the majority (~75%) of cases express oestrogen receptor (ER) and progesterone receptor (PR).^1^ ER-targeted therapies are the mainstay for treatment of this disease but drug resistance is common and usually ultimately fatal.^2^ Current standard of care for advanced ER+ breast cancer includes combination endocrine therapy with cyclin-dependent kinase 4/6 (CDK4/6) inhibition, a strategy that leads to significant improvements in progression-free survival and overall survival.^3^ However, inevitable emergence of breast cancers that are both endocrine and CDK4/6 inhibitor resistant is a major concern.^4,5^ Thus endocrine resistance remains a key clinical problem and there is a need to identify novel strategies to prevent, delay, or reverse/overcome resistance to endocrine therapy.

PR is used clinically as a biomarker of ER transcriptional activity. While several studies have shown that activated PR can antagonise ER, emerging evidence suggests an independent role for PR as a context-dependent driver of advanced ER+ breast cancer phenotypes associated with tumour progression in vitro and in vivo.^6–8^ In ER+ breast cancer cells, reversible posttranslational modifications such as phosphorylation, ubiquitinylation, and SUMOylation create unique PR species. PRs are heavily phosphorylated by mitogenic and stress-sensing protein kinases that are frequently elevated and activated in breast tumours resulting in altered transcriptional activity and target gene selection.^9–12^ We previously demonstrated that PR, when phosphorylated on N-terminal amino acid residue Ser294, enhanced the expression of gene sets associated with Her2 signalling, endocrine resistance,^13^ and embryonic and cancer stem cell (CSC) biology,^14^ including members of the insulin-like growth factor (IGF) signalling pathway. Numerous members of the IGF signalling pathway are also regulated by p-Ser294 PR in the absence of ligand.^13^ Experimental point mutation of Ser294 to Ala (S294A) blocked CD44+/CD24−/ALDH+ CSC expansion.^15^ Recently, PR, but not ER, was demonstrated to regulate a genetic programme unique to disseminating tumour cells derived from early mammary lesions arising in BALB-NeuT mice.^16^ PR-induced factors RANKL and Wnt1 (i.e. known PR target genes) substituted for PR-driven actions in these models. These studies underscore the importance of understanding the unique role of phospho-PRs in the progression of ER+ breast cancer.

The IGF/insulin signalling pathway has been implicated in the proliferation, migration, and survival of diverse human malignancies.^17,18^ Upon ligand binding, the type I IGF receptor (IGF1Rβ), insulin receptor (IR), and hybrid IGF1Rβ/IR receptors recruit insulin receptor substrate (IRS) adapter proteins to transduce downstream signalling. In breast cancers, IRS-1 and IRS-2 are the two major isoforms mediating IGF/insulin signalling; IRS-1 promotes tumour growth, whereas IRS-2 stimulates motility.^19–22^ Despite initial optimism, clinical trials targeting IGF1Rβ have been disappointing. Additionally, recent trial results show IR (namely, IR-A) is more commonly expressed in endocrine-resistant breast cancer, while IGF1Rβ expression is low/lost.^23^ Although IR expression in cancer is well documented, IR inhibition has been intentionally avoided because of concern over disrupting glucose homoeostasis relevant to diabetes risk. However, targeting key signalling pathways downstream of both receptors, including the IRS adapter proteins, represents an appealing therapeutic strategy. IGF system crosstalk with steroid hormone receptors including ER and PR is extensive; IGF1Rβ enhances ER transcriptional activity^24^ and IRS-1 is a known ligand-independent (LI) PR-B target gene.^19,25^ We previously demonstrated ER, PR-B, and IGF1Rβ cooperation within transcriptional complexes at a subset of E2-regulated target genes.^26^ These data provide a strong rationale for further study of phospho-PR interaction with the IGF pathway in ER+ breast cancer models. Herein we hypothesise that molecular crosstalk between phospho-PR and IRS-1 mediates CSC behaviour and the emergence of endocrine resistance, properties of advanced breast cancer that are relevant to recurrent metastatic disease.

## Methods

### Public data mining

Data sets were analysed using SurvExpress (http://bioinformatica.mty.itesm.mx:8080/Biomatec/SurvivaX.jsp).^27^ Analysis of grouped phospho-PR gene signature^14^ expression was performed on The Cancer Genome Atlas breast cancer recurrence data set (downloaded February 2019) and includes all patients (ER+ and ER−).

### Patient-derived xenograft (PDX) model of metastasis

NOD/SCID/ILIIrg−/− (NSG) mice were bred in house in specific pathogen-free housing with standard light/dark cycle with a maximum of 5 mice/cage. All of these studies used female mice at 6–8 weeks of age at experiment initiation. All animal experiments were reviewed and approved by Institutional Animal Care and Use Committee (IACUC) and all animals were handled in accordance with institutional IACUC and Association for Assessment and Accreditation of Laboratory Animal Care guidelines (IACUC protocol #00160) and performed under anaesthesia (isoflurane 5% induction, 2% maintenance). Breast cancer PDX were derived in female NSG mice as previously described.^28–30^ PDX lines used in these studies were: UCD4 (invasive ductal carcinoma (IDC), derived from a pleural effusion) and UCD65 (IDC, lymph node metastasis). PDX were transduced with luciferase-gfp lentivirus,^31^ regrown into tumours, and processed into single-cell suspensions as described.^31^ In all, 1 × 10^6^ viable cells suspended in 0.1 mL phosphate-buffered saline (PBS) were injected into the aorta of female NSG mice (*n* = 10/PDX line). For subcutaneous tumours, cells were placed into mammary fat pads suspended in 50% Dulbecco’s modified Eagle’s medium (DMEM)/50% Cultrex (*n* = 10/PDX line). All animals were supplemented with subcutaneous silastic pellets containing 17β-oestradiol.^28^ Animals were sacrificed by CO_2_ asphyxiation followed by cervical dislocation at 2–3 months post-injection or when moribund. At necropsy, animals were visualised for fluorescent lesions using an Olympus fluorescent stereo microscope system.

### Immunohistochemistry (IHC)

IHC was performed as previously described.^28^ Briefly, 10 μm paraffin sections were subjected to antigen retrieval in citrate buffer. Primary antibodies used were as follows: ERα (SP1, ThermoFisher, 1:100), PR (1294, DAKO, 1:500), CK5 (NCL-L-CK5, Leica Biosystems, 1:500), CK8/18 (NCL-L-5D3, Leica Biosystems, 1:500), and CA2 (SAB2700301, Sigma, 1:1600). Secondary antibodies were anti-Rabbit Ig MP-7401 (Vector, 1:2000; Burlingame, CA, USA) or anti-Mouse Ig MP-7402 (Vector, 1:2000). Development used the ImPACT DAB Peroxidase Substrate Kit (Vector). Images were captured using the Aperio Digital Pathology system (Leica Biosystems) and assembled in Adobe Photoshop CC with minimal linear changes to contrast. Quantification of the percentage of positive cells was performed using the Imagescope software (Leica) that used algorithms tuned for nuclear or cytoplasmic staining.

### General reagents

Oestradiol (E2), R5020, doxycycline hyclate (Dox), and tamoxifen were purchased from Sigma Aldrich (St Louis, MO, USA), and NT157 was purchased from Fisher Scientific (#HY-100037; Hampton, NH, USA). 83-7 monoclonal antibody (mAb) against the IR was made in house (Dr. Douglas Yee, University of Minnesota).

### Cell culture

T-47D empty vector (EV), PR-B (full-length PR), PR-B S294A, and PR-B K388R^25^ cells were cultured in Minimum Essential Medium (MEM; Corning, New York, NY, USA) containing 5% foetal bovine serum (FBS), 1% penicillin–streptomycin, 1× MEM nonessential amino acids (Life Technologies, Carlsbad, CA, USA), 6 ng/mL of insulin, and 0.2 mg/mL G418 sulfate (Corning). T-47D TamR variants were cultured as above with 1000 nM 4-hydroxytamoxifen. MCF-7 models were cultured in modified improved MEM (IMEM; Life Technologies) containing 5% FBS, 1% penicillin–streptomycin, and 67.5 ng/mL of insulin. MCF-7 cells expressing inducible IRS-1 knockdown were cultured as above with 2 µg/mL Dox for 48 h to induce knockdown. For experiments with hormone treatment, cells were hormone-starved in phenol-free modified IMEM containing 5% DCC (dextran-coated charcoal-stripped FBS; HyClone Laboratories, Logan, UT, USA) and 1% penicillin–streptomycin for 16 h prior to treatment or in complete starvation media (IMEM, 1% penicillin–streptomycin) for experiments with growth factor treatment (e.g. insulin). MCF-7 TamR cells were cultured in phenol red-free IMEM, 5% DCC, 1% penicillin–streptomycin, 67.5 ng/mL of insulin, and 100 nM 4-hydroxytamoxifen. All cell lines were confirmed *Mycoplasma* negative (2019) and authenticated by ATCC within the past 5 years.

### Stable cell line generation

MCF-7 cells were transfected per the manufacturer’s instructions with human-specific PR double nickase constructs (#sc-400198-NIC; Santa Cruz Biotechnology, Dallas, TX, USA) to knock out endogenous PR. Pooled populations were selected in and maintained as described above with 0.5 µg/mL puromycin (Fisher Scientific). Stable models of PR re-expression were generated by transfecting MCF-7 PR knockout cells with pLenti CMV-neo vector (which also served as vector control) containing wild-type (wt) PR-B, S294A PR-B, or K388R PR-B using FuGENE HD (Roche). Stable pool populations were selected in 0.6 mg/mL G418 (Corning) and maintained as described above with 0.2 mg/mL G418 sulfate. Stable shPR-expressing cell lines were created by transducing MCF-7 and TamR MCF-7 models with pLKO.1 lentiviral vectors containing target short hairpin RNA sequences TRCN0000003321 and TRCN0000003324. Pooled populations were selected in and maintained as described above with 0.5 µg/mL puromycin.

### Soft agar assays

Cells were seeded (4 × 10^4^ cells/well) in 1× sterile low melt agarose (Life Technologies) containing tamoxifen (100 nM) or insulin (0, 1, 5, or 10 nM). Soft agar assays were allowed to proceed for 21 days at 37 °C and then counted with ImageJ (Wayne Rasband, http://rsbweb.nih.gov/ij/, National Institutes of Health, Bethesda, MD, USA).

### Immunoblotting

Cells were harvested in RIPA-lite lysis and resolved using sodium dodecyl sulfate-polyacrylamide gel electrophoresis as previously described.^15^ Immunoblotting was performed with the following antibodies: PR (#sc-166169; Santa Cruz Biotechnology), pS294 PR (made in-house), IGF1Rβ (#3027 S; Cell Signaling Technology, Danvers, MA, USA), IR (#sc-57342; Santa Cruz Biotechnology), IRS-1 (Millipore #06-248), FOXO1 (#2880 S; Cell Signaling Technology), GAPDH (Santa Cruz Biotechnology #0411), goat anti-rabbit IgG-HRP (BioRad), and goat anti-mouse IgG-HRP (BioRad). Blots were developed using an ECL reagent (Super Signal West Pico PLUS; Pierce, Waltham, MA, USA) and imaged by film. Densitometric quantification of protein levels was measured in ImageJ.

### Tumoursphere assays

Tumourspheres were generated as previously described.^14,15^ ALDEFLUOR Assay Kit (Stem Cell Technologies, Vancouver, Canada) was used to assay aldehyde dehydrogenase (ALDH1) activity. Cells were plated in ultra-low attachment plates and grown in DMEM/F12 media (described above) to generate tumourspheres. Tumourspheres were collected and dissociated using StemPro Accutase (Life Technologies) and then processed according to the manufacturer’s instructions. Flow cytometry was performed on the BD LSRII H4760 flow cytometer (BD Biosciences, San Jose, CA, USA). For CD44/CD24 detection, tumourspheres were collected and dissociated as described previously^15^ and analysed by flow cytometry using APC-CD44 (1:20; BD Biosciences) or CD24-PE (1:50; BD Biosciences) conjugated antibodies.

### Real-time quantitative PCR (RT-qPCR)

Total RNA was extracted from cell samples using TriPure Isolation Reagent (Roche) and isopropanol precipitation. RNA integrity was confirmed by A260/280 ratio (>1.9) and 1000 ng was reverse transcribed to cDNA according to the manufacturer’s instructions using qScript cDNA SuperMix (Quanta BioSciences, Beverly, MA, USA). qPCR was performed using Light Cycler FastStart DNA Master SYBR Green I (Roche) on a Light Cycler 480 II Real-Time PCR System (Roche). Cycling conditions are described in Supplementary Methods. Target gene levels were normalised to 18s housekeeper gene. Primer sequences are listed in Supplementary Table S1.

### Intracellular staining and flow cytometry

Cells were seeded at 1 × 10^6^ cells in either two-dimensional (2D) adherent conditions with regular growth media or three-dimensional (3D) ultra-low attachment dishes in tumoursphere media (see above). Adherent cells were collected with trypsin and washed with 1×PBS with 1% FBS. 3D tumourspheres were collected, incubated with Accutase (Stem Cell Technologies) for 5 min at 37 °C to achieve a single-cell suspension, and then washed with 1×PBS and 1% FBS. The cells were fixed with 3% formaldehyde at room temperature (RT) for 10 min and permeabilised with 90% ice-cold MeOH for 30 min on ice. Equal numbers of cells were stained with pS294 PR antibody (1:50) for 60 min at RT, followed by Alexa Fluor 594-conjugated goat anti-rabbit IgG (Invitrogen) for 30 min at RT while protected from light. The fluorescence intensities were collected for each sample on the BD LSRII H4760 flow cytometer (BD Biosciences) and analysed using the FlowJo software (BD Biosciences). Gates were drawn based on unstained single cells, and samples stained with only the primary antibody (no secondary) were included as a negative control.

### Chromatin immunoprecipitation (ChIP) assays

Cells were fixed, harvested, and lysed according to optimised manufacturer’s instructions using the ChIP-IT Express Magnetic Chromatin Immunoprecipitation Kit (Active Motif, Carlsbad, CA, USA). Samples were homogenised using a Bioruptor sonicator (Diagenode Inc., Seraing, Liege, Belgium). ChIP reactions (100 µL isolated chromatin) were incubated with 2 µg of PR antibody or 2 µg of IRS-1 antibody overnight at 4 °C with rocking. Normal mouse IgG (Santa Cruz Biotechnology) was used in control samples. Samples were washed, eluted, reverse crosslinked, and treated with Proteinase K according to the manufacturer’s instructions. DNA was analysed by RT-qPCR as described above using primers spanning the distal promoter of the cathepsin D (*CTSD*) gene (Supplementary Table S2).

### Proximity ligation assay (PLA)

T-47D cells were plated in 8-well chamber slides and fixed with 4% paraformaldehyde for 15 min at RT. Slides were permeabilised (0.1% Triton X-100, 1×PBS) for 15 min and then processed using the Duolink Red Mouse/Rabbit Kit (Sigma) in line with the manufacturer’s protocol. Slides were incubated with primary antibody (1:250–1:1000) overnight at 4 °C. Images were taken by confocal microscopy (×40) and the ratio of puncta/nuclei for each experimental condition was calculated by manual counting of at least five images per group.

### MTT assay

MCF-7 parental and TamR cell proliferation after PR silencing was measured via MTT (3-[4,5-dimethylthiazol-2-yl]-2,5-diphenyltetrazolium bromide) assays by seeding cells in a 24-well plate in normal media (see above). At days 0 and 5, 60 µL of MTT was added per well for a final concentration of 5 mg/mL, after which the plate was incubated for 2 h at 37 °C. At this point, the medium was removed, and the cells were solubilised with 90% v/v dimethylsulfoxide/PBS. Absorbance was measured in a plate reader at 570 nm. Sample means were normalised to day 0.

### Statistical analysis

Data were tested for normal distribution using Shapiro–Wilk normality test and homogeneity of variances using Bartlett’s test. Unless otherwise stated, all results are presented as mean ± S.E.M. Statistical analyses were performed using one-way or two-way analysis of variance in conjunction with multiple comparison tests or Student’s *t* test. Significance was determined with 95% confidence.

## Results

### Phospho-PR target genes correlate with poor outcome in clinical breast cancer

**Fig. 1:**
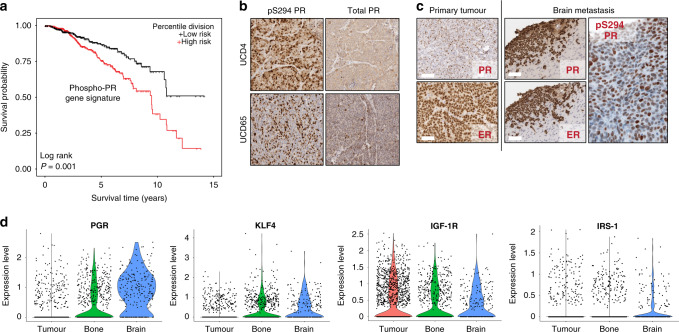
Phospho-S294 PR is enriched in PDX metastases. **a** SurvExpress was used to examine the prognostic index of patients in the TCGA Invasive Breast Cancer cohort based on the combined expression of the phospho-PR gene signature previously described by Knutson et al.^13^ **b**, **c** ER, PR and pS294 PR staining was performed on primary UCD65 and UCD4 PDX tumours and UCD65 metastases. **d** scRNAseq expression analysis of known phospho-PR target genes in primary UCD65 tumours and metastatic bone or brain lesions.

We previously identified distinct 151-gene ligand-dependent and 92-gene ligand-independent (LI) phospho-PR-specific gene signatures that were both associated with human epidermal growth factor-2 (ERB2/Her2)-positive luminal breast cancers as defined by Oncomine concepts.^13^ In a meta-analysis of the Loi data set (*n* = 377), patients whose tumours expressed both activated PR metagenes experienced early metastasis and shortened survival relative to patients lacking these metagenes.^13^ To generate a consolidated gene signature representative of elevated PR transcriptional activity, we data mined our 151 gene set and focussed on genes that were upregulated >1.5-fold upon progesterone stimulation in cells expressing a transcriptionally hyperactive form of deSUMOylated (K388R) PR-B compared with cells expressing wt PR-B. Mitogen-activated protein kinase (MAPK)-dependent phosphorylation of PR-B Ser294 attenuates Lys388 SUMOylation (i.e. typically a repressive modification of steroid receptors). Thus K388R faithfully mimics de-repressed phosphorylated PR-B (i.e. hyperactive PR) with regard to target gene selection.^13,32^ This resulted in a list of 29 K388R PR target genes (Supplementary Table S2). We further filtered out 13 genes that were K388R-PR induced but unresponsive to the PR antagonist Onapristone (Ona), resulting in a list of 16 putative phospho-PR target genes^13^ (Supplementary Table S2). To more robustly interrogate publicly available data sets, we investigated the predictive value of this newly defined “activated PR” 16-gene signature with regard to breast cancer patient outcomes. Interestingly, expression of the 16-gene signature was strongly correlated with decreased relapse-free survival (Fig. 1a).

To further explore phospho-PR activity in metastatic breast cancer, we employed PDX models established from metastatic ER+/PR+ breast cancer patients.^30^ ER expression and total or phospho-PR expression were confirmed by IHC using specific monoclonal (total) and previously characterised^14^ custom-made polyclonal (pS294 PR) antibodies (Fig. 1b, Supplementary Fig. S1). We next assessed total ER, PR, and phospho-PR expression in metastatic lesions from NSG mice PDX recipients. Since PDX tumours inefficiently metastasise in NSG mice, we established metastatic lesions by dissemination of UCD65 PDX cells into circulation. UCD65 cells primarily disseminated to the bone and brain, while UCD4 cells colonised in the liver. UCD65 brain (Fig. 1c) metastases displayed positive staining for both ER and PR, as well as phospho-PR. Furthermore, UCD4 liver metastases contained abundant phospho-PR (Supplementary Fig. S1). Single-cell (sc) RNAseq of UCD65 primary tumours and metastases revealed that *PGR* expression was enriched in bone and brain metastatic lesions relative to primary tumours (Fig. 1d). Ingenuity pathway analysis predicted *PGR* pathway activation in all three sites (not shown). Interestingly, the PR target gene *KLF4*, a known stemness factor, was also enriched in the metastatic transcriptome, along with four genes (*PHLDA1*, *NDRG1*, *TSC22D1*, *PTGES*) from the phospho-PR gene signature (not shown). The IGF signalling pathway was also significantly represented, as found in our previously reported LI phospho-PR gene signature.^26^ Notably, we observed a decrease in  known ER/PR interactor, *IGF1Rβ*, in brain metastatic lesions relative to primary PDX tumours, while levels of the IGF system adapter protein *IRS-1* were unchanged in bone lesions but modestly elevated in brain lesions. Together, these data suggest that total and phospho-PR are abundant in metastatic lesions that arise from PR-low primary tumours. Furthermore, the finding that phospho-PR target genes are elevated in metastases relative to primary tumours, suggests that these phospho-PR species are transcriptionally active and contribute to aberrant gene expression that is associated with both poor outcome and survival/outgrowth of breast cancer metastatic lesions.

### Phosphorylated PR-B confers insensitivity to tamoxifen

**Fig. 2:**
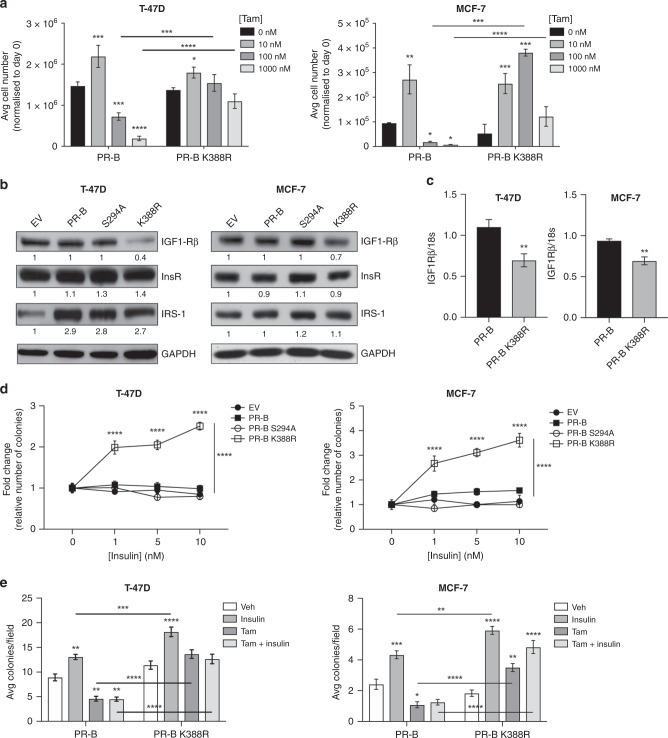
Phospho-PR promotes tamoxifen insensitivity in ER+ breast cancer models. **a** T-47D and MCF-7 wt PR-B and PRB-K388R cell growth in tamoxifen as measured by manual counting of the cells after 5 days in culture. Cell number was normalised to day 0. **b** Protein and **c** mRNA expression of the IGF1-R system. **d** Colony formation of T-47D and MCF-7 cells expressing empty vector (EV), wt PR-B, phospho-mutant PR-B (PRB-S294A), or hyperactive PR-B (PRB-K388R) grown in insulin-containing soft agar. **e** Soft agar colony formation of wt PR-B and PRB-K388R cells grown in the presence of insulin (10 nM) and tamoxifen (Tam; 100 nM). Error bars are S.E.M., *n* = 3 biological replicates; *P < 0.05; **P < 0.01; ***P < 0.001; ****P < 0.0001.

One proposed aetiology of acquired endocrine resistance is the gradual expansion of cancer cell populations that are insensitive to ER antagonists.^33,34^ To test the contribution of PR phosphorylation to acquired tamoxifen resistance, we used naturally occurring PR-null/low variants of ER+ T-47D and PR knock-out MCF-7 breast cancer cells and stably reintroduced either unmodified PR-B or transcriptionally hyperactive (deSUMOylated; K388R) PR-B. When these models were exposed to low, medium, and high doses of tamoxifen, low-dose tamoxifen (10 nM) was proliferative in both PR-B and PR-B K388R (hyperactive PR) expressing cells (Fig. 2a). However, higher concentrations of tamoxifen (100–1000 nM) were anti-proliferative in cells expressing wt-PR-B but not K388R PR-B (Fig. 2a), suggesting that unchecked PR Ser294 phosphorylation promotes a tamoxifen-insensitive state.

Tamoxifen-resistant cell lines lose expression of the IGF1Rβ and consequently become hypersensitive to insulin treatment.^35,36^ To determine whether this phenotype is mirrored in PR-B K388R models, we examined IGF1Rβ expression in T-47D or MCF-7 cells expressing empty vector control (EV), wt PR-B, phospho-mutant PR-B (S294A), or SUMO-deficient/phospho-mimic PR-B (K388R). Consistently, IGF1Rβ expression is decreased at the protein and transcript levels in breast cancer cells expressing K388R PR-B relative to other PR species (Fig. 2b, c). This result mirrors our observations in phospho-PR+ metastatic lesions (Fig. 1d). Notably, IRS-1 levels increased in PR+ T-47D models relative to PR-null EV controls, while IRS-1 levels remained similar across wt, S294A, and K388R T-47D models; manipulation of PR had little effect on IRS-1 levels in MCF-7 cells. We next tested colony formation in increasing concentrations of insulin and observed that PR-B K388R cells (both T-47D and MCF-7 models) displayed greatly increased sensitivity to insulin-driven proliferation relative to EVs, as well as cells expressing either wt PR-B or S294A PR-B (Fig. 2d). Furthermore, PR-B K388R models remained insensitive to tamoxifen in the presence of insulin (Fig. 2e). Together, these data implicate phospho-PR crosstalk with IR signalling in the development of tamoxifen insensitivity.

### Phospho-PR-B and IRS-1 are preferentially co-recruited to E2-regulated genes

**Fig. 3:**
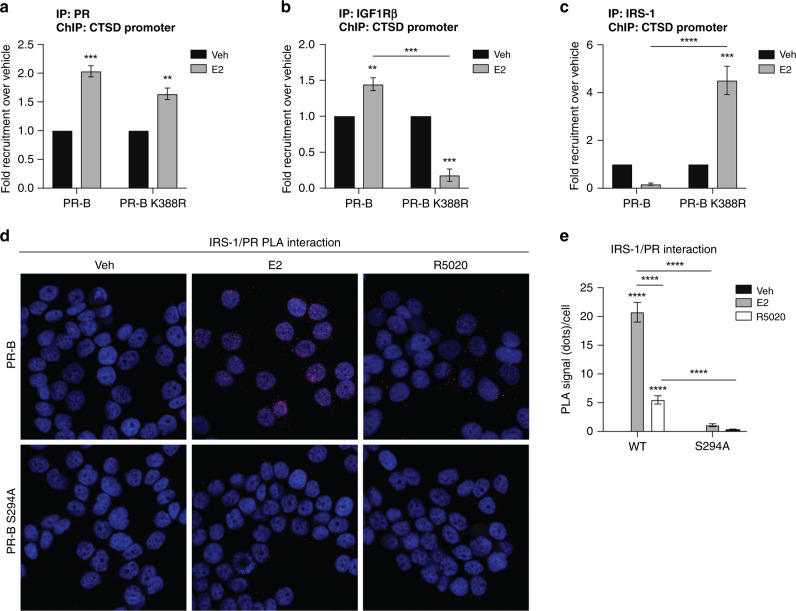
PR preferentially interacts with IRS-1 at ER/PR target genes when PR is phosphorylated. **a** PR, **b** IGF1-Rβ, and **c** IRS-1 ChIP to the *CTSD* promoter in T-47D PR-B and PRB-K388R cells treated with 1 nM E2. Data are presented as fold recruitment over vehicle of combined *n* = 4 experiments (technical replicates). **d** Proximity ligation assay showing an interaction between PR and IRS-1 in T-47DT-47D cells expressing wt PR-B or phospho-mutant PR-B (PRB-S294A) treated with 1 nM E2 or 10 nM R5020. Puncta were counted in ImageJ and quantified (**e**). Error bars are S.E.M.; *n* = 3 biological replicates; **P < 0.01; ***P < 0.001; ****P < 0.0001.

We previously reported a requirement for PR-B expression and the presence of ER/PR/PELP1 and ER/PR/IGF1Rβ complexes at specific steroid receptor target genes, including *CTSD*^26^ in response to oestrogen stimulation; CTSD expression is associated with increased risk of relapse and metastasis.^37^ Given PR-B K388R cells lost the expression of IGF1Rβ, we asked whether either the IR or IRS-1 instead bind to the *CTSD* promoter in ER+ breast cancer cells expressing either wt or hyperactive K388R PR-B. Notably, E2-stimulated recruitment of K388R PR-B to the *CTSD* promoter was comparable to that of wt PR (Fig. 3a). Similar to our previous results,^26^ IGF1Rβ was co-recruited to the same *CTSD* promoter region in ER+ cells expressing wt PR-B. Unsurprisingly, IGF1Rβ recruitment was completely abrogated in PR-B K388R cells (Fig. 3b). While IR was not detected at these promoter regions (data not shown), IRS-1 recruitment to the ER/PR *CTSD* promoter site was strikingly increased in cells expressing K388R PR-B relative to cells expressing wt PR-B (Fig. 3c). These data suggest a switch in the regulation of phospho-PR target genes by ER/pS294-PR/IRS-1 complexes when IGF1Rβ is lost during the acquisition of tamoxifen resistance.

Functional interactions between steroid receptors and cytoplasmic signalling pathways (i.e. ER/PR/c-Src complexes) are well documented. Previous studies showed that IRS-1 and ER formed a complex in the cytosol.^38^ We therefore probed the location of IRS-1/PR-containing complexes by PLA in ER+ T-47D PR-B (wt) and PR-B S294A mutant breast cancer models. Cells expressing either wt or S294A PR were treated with either vehicle or E2 and subjected to PLA using specific antibodies. While no PR/IRS-1 complexes were detected in vehicle-treated cells, E2 treatment strikingly induced the appearance of IRS-1/PR-positive puncta that were primarily located in the nucleus (Fig. 3d, e). Fewer puncta were observed in the cytosol of E2-treated cells (Fig. 3d, e), suggesting that PR/IRS-1 complexes exist in both subcellular compartments but are retained in the nucleus. Notably, PR/IRS-1 complexes were significantly reduced in cells expressing S294A phospho-mutant PR-B and these puncta appeared to be restricted to the cytoplasm (Fig. 3d, e). Interestingly, in contrast to E2 treatment, progestin (R5020) treatment increased the appearance of PR/IRS-1 puncta that were primarily cytoplasmic (Fig. 3d, e). Taken together, our data suggest that pSer294-PR and IRS-1 interact upon either E2 or progestin treatment but are specifically co-recruited to ER/PR target genes such as *CTSD* in the presence of E2.^26^ Our studies nominate IRS-1 as a partner and potential therapeutic target in ER/PR complexes at E2-regulated genes that require both ER and PR (but not progestins) for their expression.

### Pharmacological inhibition/knockdown of IRS-1 reduces CSC outgrowth

**Fig. 4:**
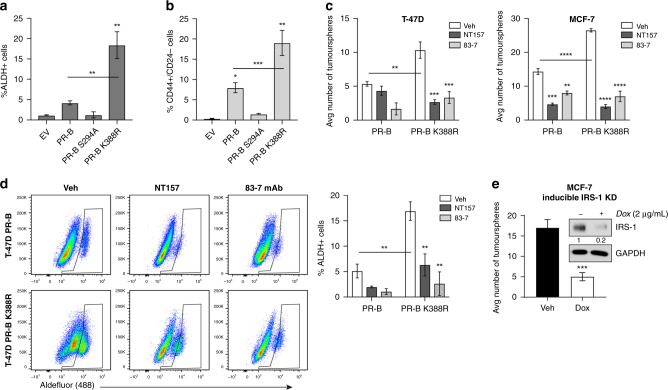
IRS-1 inhibition decreases tumoursphere formation. **a** ALDH1 activity was determined using the Aldefluor assay in T-47D tumourspheres. **b** CD44+/CD24− populations in T-47D tumourspheres were analysed by flow cytometry. **c** The effect of NT157 (IRS-1 inhibitor; 3 µM) and 83-7 monoclonal antibody (insulin receptor degrader; 5 µg/mL) treatment on tumoursphere number and **d** ALDH1 activity was examined. **e** Tumoursphere formation was examined after inducible IRS-1 knockdown. Inset confirms Dox-induced knockdown of IRS-1 protein by western blot. Graphed data are mean ± S.E.M. of combined *n* = 4 independent experiments (technical replicates). *P < 0.05; **P < 0.01; ***P < 0.001; ****P < 0.0001.

Previous studies indicated that cells expressing hyperactive PR-B (K388R) form increased primary and secondary tumourspheres relative to cells expressing wt PR-B.^14^ Herein we extended this finding to measure ALDH1 activity and the relative populations of CD44+/CD24− cells, common markers of breast CSCs.^39,40^ Both ALDH+ and CD44+/CD24− populations were significantly increased in K388R PR-B+ tumourspheres (Fig. 4a, b and Supplementary Fig. S2), confirming that phospho-PR species enable robust breast CSC outgrowth in vitro. Given our data linking phospho-PR-B and IRS-1, we next investigated whether pharmacologically targeting the IR/IRS-1 pathway abrogates CSC outgrowth in PR+ breast cancer models. Thus we tested both an mAb specific for IR (83-7) as well as an IRS-1 degrader (NT157) in tumoursphere assays. In T-47D models, NT157 and 83-7 were ineffective in parental wt PR-B+ cells, which exhibit low basal tumoursphere formation as scored by manual counting of tumourspheres >100 µm.^14,15^ However, both agents significantly reduced PR-B K388R (hyperactive PR/IGF1Rβ-low) tumoursphere formation (Fig. 4c). Similarly, IRS-1 inhibition and IR degradation effectively blocked tumoursphere formation of T-47D and MCF-7 cells expressing either wt PR-B or PR-B K388R (Fig. 4c). Furthermore, NT157 and 83-7 treatment reduced ALDH1 activity as measured by flow cytometry (Fig. 4d). The requirement for IRS-1 in tumoursphere formation was directly tested using a model of Dox-inducible IRS-1 knockdown in ER+/PR+ MCF-7 cells (Fig. 4e). These data provide the first in vitro evidence that IRS-1 and IR targeting are useful strategies with which to block stemness phenotypes mediated by phospho-PR species in ER+ breast cancer cells.

### PR expression is required for TamR tumoursphere formation

**Fig. 5:**
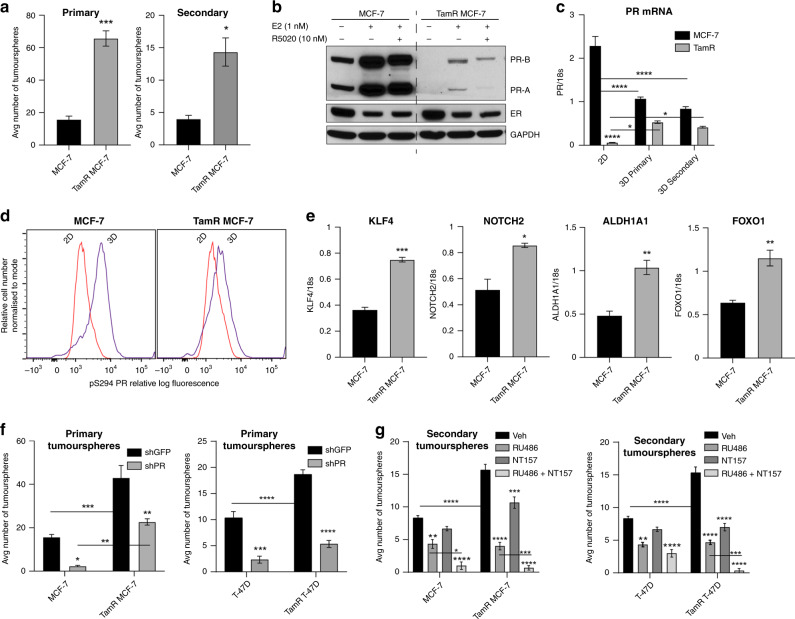
PR expression is necessary for tumoursphere outgrowth in an endocrine-resistant model of ER+ breast cancer. **a** Primary and secondary tumoursphere formation of parental and tamoxifen-resistant (TamR) MCF-7 cells. **b** Protein expression of PR and ER in TamR MCF-7 cells pretreated with 1 nM E2 for 48 h, followed by acute R5020 treatment (1 h; 10 nM). **c** PR transcript levels were analysed in cells grown in adherent 2D conditions, as 3D primary or 3D secondary tumourspheres using RT-PCR analysis. **d** Single-cell phospho-PR levels were measured in 2D adherent cultures and 3D tumourspheres by phospho-specific flow cytometry. Flow plots are representative of *n* = 3 independent experiments. **e** *KLF4*, *NOTCH2*, *ALDH1A1*, and *FOXO1* mRNA levels were measured by RT-PCR. **f** PR was silenced in endocrine refractory MCF-7 and T-47D cells using shRNA and tumoursphere formation tested. **g** Secondary tumoursphere formation was examined with Mifepristone (RU486; 100 nM) and/or NT157 (3 µM) treatment in MCF-7 and T-47D parental and TamR models. Error bars are S.E.M.; *n* = 3 biological replicates; *p < 0.05; **P < 0.01; ***P < 0.001; ****P < 0.0001.

We next examined the role of pSer294-PR in well-defined models of acquired tamoxifen resistance.^35,36^ Consistent with previous observations, TamR MCF-7 cells form increased tumoursphere numbers compared to parental MCF-7 cells (Fig. 5a).^41^ Notably, PR expression in TamR models carried in vitro (2D) is typically undetectable by western blot in the absence of E2 pretreatment (i.e. E2 is needed to induce expression of endogenous PRs; Fig. 5b). However, PR mRNA is upregulated when TamR cells are grown in 3D culture conditions (Fig. 5c). To quantitatively detect PR phosphorylation levels in these PR-low/null models, we employed flow cytometry using pS294 PR labelling of fixed, permeabilised cells.^42,43^ The advantage of this method is that phospho-PR species may be detected and quantified even when expressed in a minority subpopulation.^42,43^ Relative to 2D culture conditions, parental MCF-7 cells grown in 3D tumoursphere conditions exhibited increased PR phosphorylation as shown by a rightward-shift in fluorescence intensity (Fig. 5d). TamR MCF-7 3D tumourspheres also displayed a modest but reproducible shift in fluorescence intensity relative to 2D adherent conditions (Fig. 5d). We next examined basal expression of known pSer294-PR target genes and observed a significant increase in *KLF4*, *NOTCH2*, and *FOXO1* transcript levels in TamR cells as well as increased levels of *ALDH1A1*, an enzyme known to be enriched in stem cells (Fig. 5e).^15,40^ Increased FOXO1 and IRS-1 (i.e. known PR-induced target genes) protein levels were also observed (Supplementary Fig. S3A). Together, these data support a role for low levels of hyperactivated PR in the observed increase in tumoursphere formation in these models.

To test the requirement for PR in the CSC biology of these TamR models, we used small hairpin targeting to knockdown endogenous PR levels in MCF-7 parental and TamR cell lines and extended these studies to include paired T-47D parental and TamR models (Supplementary Fig. S3B). Partial PR knockdown (~20–30% as measured by densitometry) had modest dampening effects on cell proliferation (Supplementary Fig. S3C). Strikingly however, PR knockdown significantly reduced outgrowth of both MCF-7 and T-47D TamR primary tumourspheres (Fig. 5f); we were unable to obtain secondary tumourspheres following PR knockdown (not shown). We next used combination strategies in tumourspheres derived from two TamR models (MCF-7 and T-47D). Tumoursphere formation was then measured in the absence or presence of agents targeting both IRS-1 (NT157) and PR (Ona; RU486, RU). Either IRS-1 or PR inhibition alone were effective at reducing both parental and TamR MCF-7 cell tumoursphere formation, while the combination of NT157 and Ona further reduced tumoursphere numbers in TamR MCF-7 models (Supplementary Fig. S3D). In T-47D models, which have previously been shown to express mutant p53^44^ and are reported to be insensitive to Ona,^15^ IRS-1 inhibition strikingly reduced T-47D TamR tumoursphere formation (Supplementary Fig. S3D). Notably, the combination of NT157 and mifepristone (RU) was highly effective in both models when compared to either treatment alone (Fig. 5g). Similarly, IR degradation was more effective than PR inhibition alone in both T-47D and MCF-7 TamR models (Supplementary Fig. S3E). These results indicate that despite extremely low PR levels in TamR subpopulations, hyperactive pSer294-PRs contribute to CSC biology as measured by in vitro tumoursphere assays. These data suggest that a minority subpopulation of pre-existing phospho-PR+ breast cancer cells and/or inducible PRs are capable of significantly expanding the therapy-resistant stem-like cell compartment in apparently PR-null but ER+ TamR (i.e. luminal B-type) models. These phospho-PR species are induced in anchorage-independent contexts (Fig. 5c, d) and may be relatively insensitive to currently available PR ligands (antagonists/partial agonists or selective PR modulators) but can be effectively targeted via inhibition of the IR/IRS-1 signalling pathway.

## Discussion

Improved treatments for endocrine-resistant ER+ breast cancers are urgently needed. Breast CSC activity and frequency have been shown to increase in response to Tamoxifen and Fulvestrant in ER+ patient samples and in metastatic PDX models in vivo.^33^ These occult cells are thought to be responsible for ER+ relapse. Herein we describe a mechanism underpinning the development of resistance to tamoxifen whereby phosphorylated PR-B interacts with the IRS-1 adapter protein at previously identified ER/PR target genes. Furthermore, we show that targeting PR, IRS-1, or the upstream IR abrogate secondary tumoursphere formation and reduce expression of stem-like markers. Our studies suggest that routine targeting of activated PRs as part of ER-targeted therapies may prevent relapse of ER+ tumours by disruption of ER/PR/IRS-1 complexes. Phospho-PRs or genetic markers of phospho-PR action (i.e. a scorable phospho-PR gene signature) may provide useful biomarkers for selection of patients at high risk of developing endocrine resistance who are likely to respond to combination therapies that include antagonism of PR, IRS-1, or other non-ER signalling components of this transcriptional complex. Further studies characterising these novel IRS-1/phospho-PR target genes are required.

Alterations in the IGF1Rβ/IR system signalling have been reported in models of endocrine resistance in vitro^35,36^ and here we build on this to show that this similarly occurs in both ER+/PR+ PDX (Fig. 1) and ER+/hyperactive-PR+ breast cancer models (Fig. 3). These data are further supported by meta-analyses of recurrent endocrine-treated tumours, which showed lower levels of IGF1Rβ compared with pretreated tumours.^45,46^ Our in vitro results show that insulin signalling is important in endocrine-resistant cells but less relevant to parental (i.e. endocrine sensitive) cells (Figs. 2 and 5). This is likely underpinned by IGF1Rβ/IR hybrid receptors, which allow endocrine-sensitive cancer cells to expand their ligand-binding capacity whereby insulin and IGF-1 can signal through either heterodimer or specific homodimers.^36^ In TamR cells, however, where there is little IGF1Rβ, IR becomes the predominant receptor driving insulin and IGF-stimulated growth. Combined with our data showing that IR inhibition reduces stem-like cell outgrowth, this implicates IR as an important target in endocrine-resistant tumours. However, a major concern in targeting the IR is the resulting disruption of glucose homoeostasis in normal tissues. Taken together with our results implicating IRS-1 in the development of aggressive breast cancer phenotypes (Fig. 5), IRS-1 is emerging as an attractive target as a key ER/PR-binding partner.

Using a small molecule inhibitor of IRS-1, NT157, we showed striking reduction of ER+ breast cancer tumoursphere formation and expression of stem-like markers (ALDH1, CD44/CD24; Fig. 4). In support of this, NT157 was shown to have inhibitory effects on tamoxifen-resistant ER+ breast cancer proliferation in both 2D and 3D.^18,47^ Notably, CSCs are thought to be non-proliferative; therapeutic interventions targeting proliferative cells may initially slow tumour growth but induce selective pressure allowing outgrowth of CSCs.^48,49^ Thus disruption of IRS proteins represents an ideal strategy to inhibit both hormone and growth factor-driven signalling pathways; NT157 downregulation of IRS proteins resulted in dual IGF1R and IR signalling inhibition in melanoma, prostate, and breast cancers and, as shown herein, target minority CSC populations.^47,50,51^ Furthermore, phosphoproteomic profiling of A375 melanoma cells treated with NT157 showed that multiple kinase signalling pathways are affected, including p38 MAPK, JNK, and c-Src,^52^ which are known to phosphorylate the PR N-terminus, including Ser294.^9,10,12,25^ Herein inducible knockdown of IRS-1 in MCF-7 cells phenocopied the block of CSC outgrowth observed with either PR knockdown or IRS-1 or IR inhibitors. Thus IRS-1 inhibition represents a novel strategy to target phospho-PR-specific signalling in ER+ breast cancer, particularly in the endocrine-resistant setting.

Two well-characterised ER+/PR-low PDX models derived from patients with advanced breast cancer exhibited high total and pS294 PR expression in metastatic brain and liver lesions (Fig. 1 and Supplementary Fig. S1). Surprisingly, robust phospho-PR staining was detected even when total PR levels were modest in primary tumours. This finding suggests that luminal tumour cells expressing activated phospho-PR species confer increased aggressiveness, the clinical implication being that luminal B-type breast cancers (i.e. scored as PR-low/null by IHC) may contain activated PRs. Phospho-PR+ breast cancer cell subpopulations may more successfully disseminate. This conclusion is supported by studies showing that PR downregulation is associated with a switch from proliferation to migration in a BALB-NeuT model of tumour development.^16^ Notably, we observed that phospho-PR target genes associated with breast CSC biology were also enriched in metastatic lesions indicating hyperactive PR signalling. Importantly, scRNAseq identified *PGR* and *IRS-1* enrichment in metastases compared to primary tumours, whereas *IGF1R* expression trended downward in metastases. The global transcriptomic and epigenetic consequences of phospho-PR and IRS-1 co-expression as well as the relevant PR-dependent steps within the metastatic cascade are topics of future investigation.

## Supplementary information

Supplementary Materials

## Data Availability

Raw singe-cell sequence data have been deposited in the gene expression omnibus (GEO) database (GSE131007).
