# Supplementary material for: Insulin receptor substrate-1 (IRS-1) mediates progesterone receptor-driven stemness and endocrine resistance in oestrogen receptor+ breast cancer
Source: Br J Cancer. 2020 Nov 4;124(1):217–27. doi: 10.1038/s41416-020-01094-y (PMC7782753; doi:10.1038/s41416-020-01094-y)
Supplement: Supplementary file 1 — Supplementary Materials [file 41416_2020_1094_MOESM1_ESM.docx]

**SUPPLEMENTARY METHODS**

**RNA preparation and sequencing**. For single cell RNAseq, single cell suspensions were prepared as described ^30^ from parental tumors and brain and liver metastases (1 representative mouse/group). Bone cells were isolated using a different protocol ^1^. Human PDX cells were enriched using a mouse cell depletion kit (Miltenyi Biotech). All samples not used immediately were frozen in CryoStor media (Sigma). Cells were captured using the 10X Genomics Chromium system, libraries prepared according to Chromium platform, and sequenced (>500 cells/group) using the Illumina HiSeq 4000 or NovaSe1 6000 System.

**Single-cell RNA-seq data processing in UCD65**. scRNA-seq libraries were aligned to the human (GRCh38) genomes and UMIs deduplicated using Cell Ranger 2.1.1. Cell barcodes identified as human-murine doublets were discarded. Valid cell barcodes identified using Cell Ranger were further filtered to remove low quality cells by excluding all cell barcodes either greater than 30% of UMIs derived from mitochondrial genes or fewer than 200 genes detected. Cell barcodes with more than 8000 (UCD65) detected were also removed to exclude likely human doublets. The total number of human cells used in UCD65 for downstream analysis were: primary tumor = 1502, brain metastasis = 239, bone metastasis = 725. Variable genes were detected using the vst method in Seurat 3.1 ^2^. Next, principal component analysis (PCA) was run on the variable genes and harmony  ^3^ was run in these PCs for batch correction. The first 20 PCs were investigated by elbow plot to determine the true signals and the first 15 PCs were selected for further analysis. Seurat's graph-based clustering algorithm was also run using the first 15 PCs to identify clusters of related cells in the data. The normalized gene expression data in the primary tumor, brain metastasis and bone metastasis were used for plotting the expression profiles of PGR, KLF4, IGF-1R, IRS-1.

**qRT-PCR**. Cycling conditions for qPCR were as follows: initial denature at 95°C for 10 min; 35 X denature at 95°C for 10 s, anneal at 52°C for 10 s, and extension at 72°C for 11 s.

**SUPPLEMENTARY FIGURES**

**FIGURE S1.**

**
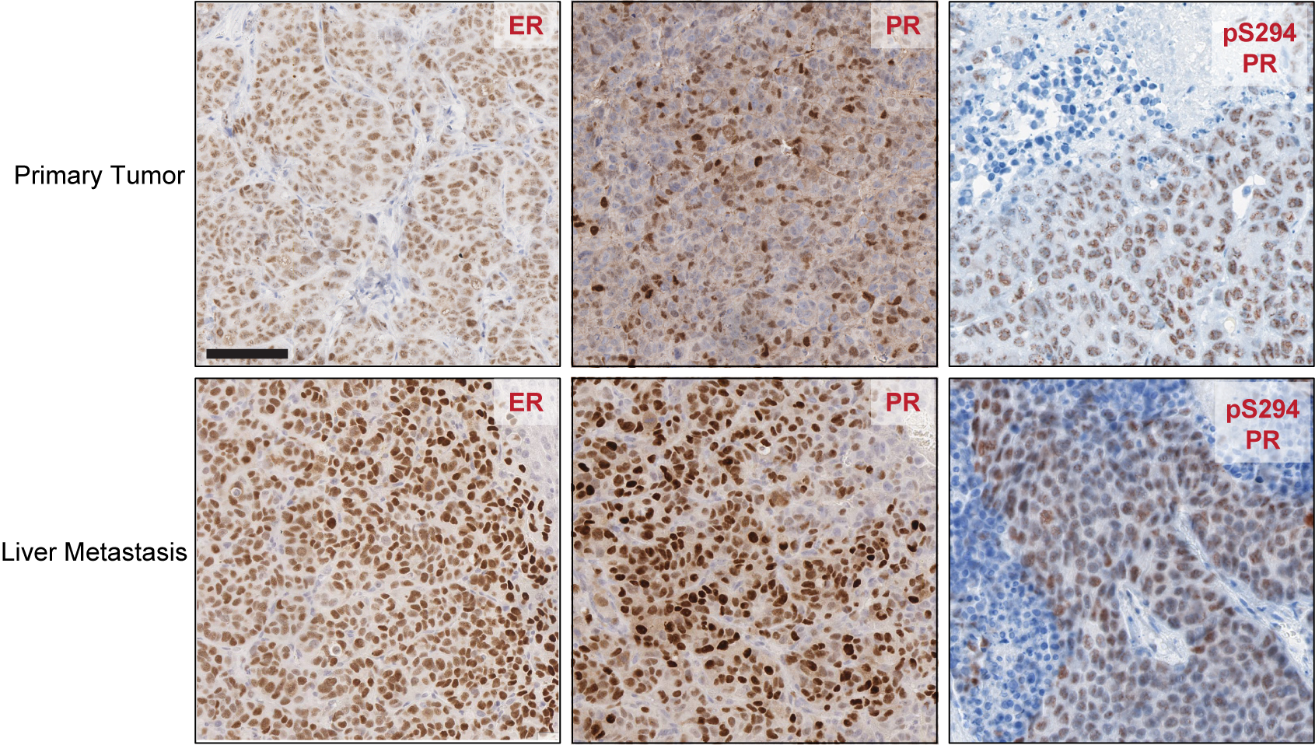
**

**Supplementary Figure S1. pS294 PR is expressed in UCD4 tumors and metastatic lesions.** Total ER, PR, and phospho S294 PR staining was performed on UCD4 PDX tumors and metastases. Scale bar = 100 um.

**FIGURE S2.**

**
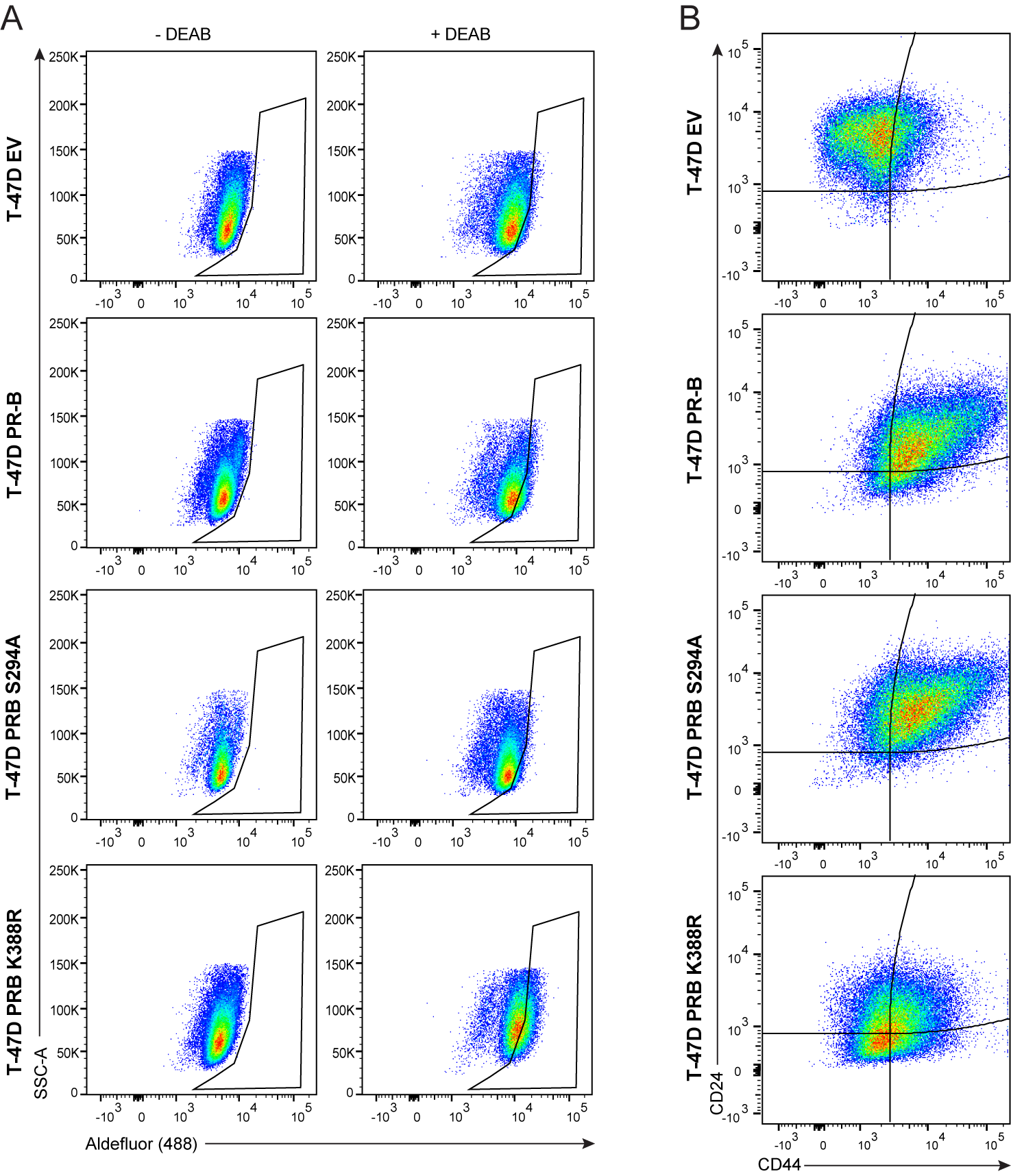
**

**16.9%**

**4.2%**

**10.6%**

**6.67%**

**0.27%**

**14.6%**

**1.31%**

**5.35%**

**Supplementary Figure S2. Representative flow cytometry scatter plots.** **A** ALDH1 activity was measured in T47D tumorspheres. Diethylaminobenzaldehyde (DEAB) inhibition of ALDH1 was included for compensation. Data are representative of n=5 independent experiments (technical replicates). **B** CD44/CD24 populations from primary T47D tumorspheres. Data are representative of n=4 independent experiments.

**FIGURE S3.**

**
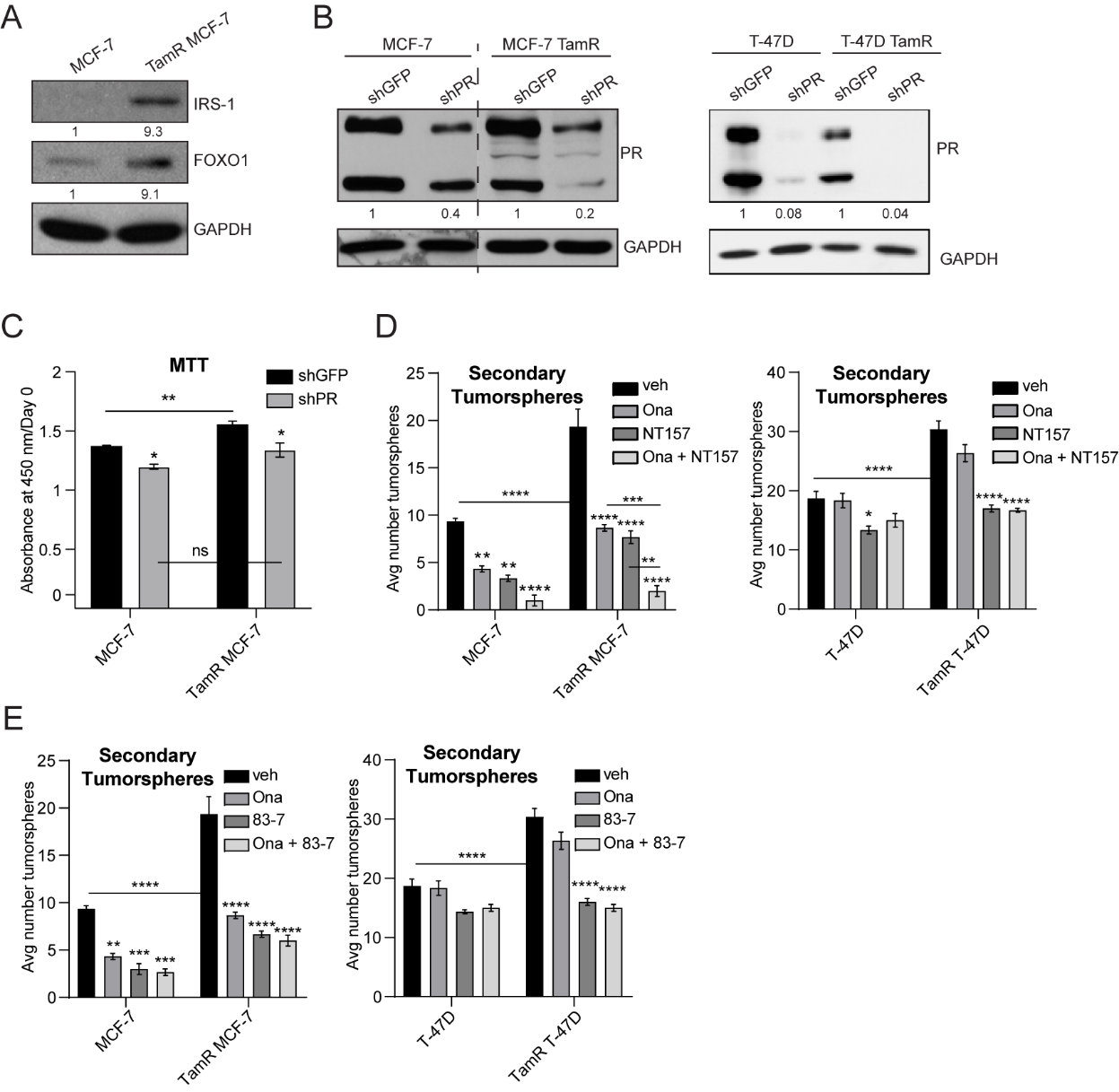
**

**Supplementary Figure S3. PR inhibition impacts tumorsphere formation. A** Basal protein levels of IRS-1 and FOXO1 in MCF-7 TamR models. **B** PR was silenced in T-47D and MCF-7 endocrine resistant models. Densitometry (indicated) confirms at least 40% knock-down. **C** Proliferation of MCF7 and TamR MCF7 cells expressing shGFP or shPR was analyzed by MTT assay. **D** Secondary tumorsphere formation was examined with Onaprisone (100 nM) and NT157 (3 µM) or **E** 83-7 mAb (5 µg/mL) treatment. Error bars are S.E.M; n=2 technical replicates each containing n=3 biological replicates; **P*<0.05; ***P*<0.01.

**SUPPLEMENTARY TABLES**

**Supplementary Table S1: Primer sequences**

| PR | Fwd | 5’ACACCTTGCCTGAAGTTTCG |
| --- | --- | --- |
|  | Rev | 5’CTGTCCTTTTCTGGGGGACT |
| IRS-1 | Fwd | 5’TCACAGCAGAATGAAGACC |
|  | Rev | 5’CTACTGATGAGGAAGATATGAGG |
| IGF1-Rβ | Fwd | 5’GCAAAGGGGACATAAACACC |
|  | Rev | 5’TGATGATGCGATTCTTCGAC |
| InsR | Fwd | 5’CAACGTGGTTTTCGTCCCC |
|  | Rev | 5’AGATGACCAGCGACTCCTTG |
| KLF4 | Fwd | 5’AGACGCGAACGTGGAGAAAG |
|  | Rev | 5’GCTGCCGAGGACCTTCTG |
| NOTCH2 | Fwd | 5’CATTGCAGTGTCGAGATGGC |
|  | Rev | 5’CCCCAAGAAGCCTTCTGGAC |
| ALDH1A1 | Fwd | 5’GCCATAACAATCTCCTCTGCTC |
|  | Rev | 5’ACTCTCCCAGTTCTCTTCCATTT |
| FOXO1 | Fwd | 5’ACGAGTGGATGGTCAAGAGC |
|  | Rev | 5’GCACACGAATGAACTTGCTG |
| 18S | Fwd | 5’GGAGAGGGAGCCTGAGAAAC |
|  | Rev | 5’TCGGGAGTGGGTAATTTGC |
| CTSD (ChIP) | Fwd | 5’CCTCCTCAACTGCTCTTGCA |
|  | Rev | 5’GCGGCTGAGATGCTGAGTCA |
|  |  |  |

**Supplementary Table S2: Phospho-PR target gene signature**

| \| ***29 gene list*** \| ***16 gene list*** \| \| --- \| --- \| \| TUBA3D \| TUBA3D \| \| TUBA3E \| TUBA3E \| \| DDC \| UTS2D \| \| SLC26A3 \| SPRYD5 \| \| UTS2D \| MAP1A \| \| AFAP1L2 \| SPRINK5L3 \| \| SPRINK5L3 \| THY1 \| \| SPRYD5 \| PDK4 \| \| MAP1A \| KIAA0513 \| \| THY1 \| MSX2 \| \| ZNF812 \| PHLDA1 \| \| PDK4 \| KLF9 \| \| KIAA0513 \| TSC22D1 \| \| ASB9 \| KHDRBS3 \| \| OGFRL1 \| SLC35C1 \| \| MSX2 \| ATG12 \| \| PHLDA1 \|  \| \| CHN2 \|  \| \| KLF9 \|  \| \| FOXJ2 \|  \| \| CBLL1 \|  \| \| SLC35C1 \|  \| \| TSC22D1 \|  \| \| KHDRBS3 \|  \| \| ZNF26 \|  \| \| PXMP4 \|  \| \| NDRG1 \|  \| \| LOC9362 \|  \| \| ATG12 \|  \| |  |
| --- | --- | --- | --- | --- | --- | --- | --- | --- | --- | --- | --- | --- | --- | --- | --- | --- | --- | --- | --- | --- | --- | --- | --- | --- | --- | --- | --- | --- | --- | --- | --- | --- | --- | --- | --- | --- | --- | --- | --- | --- | --- | --- | --- | --- | --- | --- | --- | --- | --- | --- | --- | --- | --- | --- | --- | --- | --- | --- | --- | --- | --- |

**Supplementary References**

1. Stern AR, Stern MM, Van Dyke ME, Jahn K, Prideaux M, Bonewald LF. Isolation and culture of primary osteocytes from the long bones of skeletally mature and aged mice. BioTechniques. 52(6):361-73 (2012)

2. Butler A, Hoffman P, Smibert P, Papalexi E, Satija R. Integrating single-cell transcriptomic data across different conditions, technologies, and species. Nature biotechnology. 36(5):411-20 (2018)

3. Korsunsky I, Millard N, Fan J, Slowikowski K, Zhang F, Wei K, et al. Fast, sensitive and accurate integration of single-cell data with Harmony. Nature methods. 16(12):1289-96 (2019)
